# Patterns of early post-disturbance reorganization in Central European forests

**DOI:** 10.1098/rspb.2024.0625

**Published:** 2024-09-25

**Authors:** Rupert Seidl, Mária Potterf, Jörg Müller, Monica G. Turner, Werner Rammer

**Affiliations:** ^1^ School of Life Sciences, Technical University of Munich, Hans-Carl-von-Carlowitz-Platz 2, Freising 85354, Germany; ^2^ Berchtesgaden National Park, Doktorberg 6, Berchtesgaden 83471, Germany; ^3^ Department of Animal Ecology and Tropical Biology, Biocenter, Field Station Fabrikschleichach, University of Würzburg, Glashüttenstr. 5, Rauhenebrach 96181, Germany; ^4^ Bavarian Forest National Park, Freyungerstr. 2, Grafenau 94481, Germany; ^5^ Department of Integrative Biology, University of Wisconsin-Madison, Madison, WI 53706, USA

**Keywords:** forest resilience, post-disturbance forest recovery, forest development, drought and bark beetle mortality, salvage logging, tree regeneration

## Abstract

Disturbances catalyse change in forest ecosystems, and a climate-driven increase in disturbance activity could accelerate forest reorganization. Here, we studied post-disturbance forests after the biggest pulse of tree mortality in Central Europe in at least 170 years, caused by drought and bark beetle (Scolytinae) outbreaks in 2018–2020. Our objectives were to characterize the early state of tree regeneration after mortality, quantify patterns of reorganization relative to undisturbed reference conditions and assess how management and patch size affect forest reorganization after disturbance. We surveyed 1244 plots in 120 patches under managed (salvage-logged, often planted) and unmanaged (deadwood remaining on site, no planting) conditions in Germany. We found that regeneration density on disturbed sites was high (median 11 897 stems ha^−1^), resulting from a cohort of advance regeneration. Disturbances were strong drivers of change, with indications for resilience on only 36.3% of patches. Reassembly (i.e. a change in species composition) was the dominant pattern of reorganization (61.5%), and *Picea abies* forests changed most strongly. Post-disturbance management facilitated forest change, particularly promoting a change in species composition. The strength of reorganization increased with patch size. We conclude that the recent wave of tree mortality will likely accelerate forest change in Central Europe.

## 1. Introduction

Disturbances are changing in forest ecosystems around the world. Pulses of tree mortality—here referred to as disturbance—can be caused either by natural agents such as wind, wildfire, drought and insects, or by human land use [1,2]. Natural disturbances are among the most climate-sensitive processes in forest ecosystems, and are responding strongly to the ongoing changes in the climate system [3,4]. Human activities such as the biotic homogenization of landscapes [5] and the introduction of non-native species [6] are further exacerbating change in forest disturbance regimes. While disturbances are a natural part of ecosystem dynamics and many forests are well adapted to their historical disturbance regime, the ecological responses to emerging novel disturbance regimes remain largely unknown [7].

Disturbances are catalysts of ecological change. They free resources and can break the legacy lock of pre-disturbance systems, allowing new species and structures to establish [2,8]. This holds particularly true for forest ecosystems, which are often governed by stabilizing feedbacks for decades to centuries, with change only happening after the canopy is disturbed. The period immediately following a disturbance is thus critical for forest change, as it is then when ecosystems reorganize and the potential for fundamental change is the greatest. Once a new cohort of trees has established and the canopy closes again, so does the window for change. After disturbance, forests often lock into a development trajectory for decades to centuries. Thus, quantifying patterns of reorganization is important to understand ecosystem responses to disturbance, and can give early indication of potential future trajectories of forest ecosystems [9,10].

Central Europe was a global hotspot of tree mortality in the recent past. Triggered by a multi-year drought [11], tree mortality in the years 2018–2020 reached levels that were unprecedented for at least 170 years [12,13]. The highest levels of drought mortality were recorded in Germany and Czechia. Mortality was observed across forest types, with Norway spruce (*Picea abies* (L.) Karst.) forests being particularly affected because of their high drought susceptibility [14], and because of a wave of bark beetle (*Ips typographus* L.) outbreaks triggered by drought [15]. The tree mortality pulse of 2018–2020 altered the imprint of disturbances on forests in Central Europe, with patch sizes (generally small) and patch densities (generally low) increasing considerably [12]. However, consequences of this widespread pulse of mortality on forest development remain unclear, and patterns of early post-disturbance reorganization have not been quantified to date. Based on previous work, we hypothesize that this pulse of mortality may considerably alter the structure and composition of forest ecosystems. Specifically, post-disturbance reorganization may foster the establishment of trees better adapted to emerging climatic conditions [16,17]. The alternative hypothesis is that forests in Central Europe are resilient *sensu stricto* (i.e. self-replacement of the previously dominating species and structures after disturbance [18]. Resilience could, for example, result from the widespread prevalence of advance regeneration (a cohort of regenerating trees that established prior to canopy disturbance and persist into the post-disturbance system as a biological legacy of the pre-disturbance stand). Previous studies found that advance regeneration is important for the dynamics of forests in Central Europe [19–21], and the prevalence of a cohort of trees that established already prior to disturbance could result in little to no change in forest structure and composition post-disturbance.

Following the recent wave of mortality, the fate of disturbed forests in Central Europe has been widely discussed among foresters, policy makers and the general public. While reports from other parts of the world indicate that post-disturbance regeneration is faltering [22], the response of Europe’s forests to changing disturbance regimes remains unclear. Previous analyses based on remote sensing data suggest high post-disturbance recovery potential of the forests of Central Europe [10,23], yet field-based analyses of tree densities on recently disturbed sites remain scarce. A second discussion theme concerns whether and how to treat disturbed sites. In general, salvage harvesting areas affected by natural disturbances (i.e. removing dead trees to market their timber and partly recuperate the economic losses from disturbance) and planting (at least parts) of cleared areas is the default post-disturbance management in Central Europe [24]. Yet, concerns about the ecological consequences of salvage logging are increasingly recognized also in European forestry [25]. Furthermore, the high costs of planting large areas and the lack of sufficient seedlings complicate reforestation. Therefore, leaving disturbed areas untreated (i.e. retaining deadwood on site and refraining from tree planning) has been proposed as a viable alternative for the newly created early seral stages. However, the effects of these alternative treatment options on post-disturbance tree communities remain unclear.

Here, we studied post-disturbance forest reorganization in Central Europe, focusing on the mortality pulse of the years 2018–2020. Our specific objectives were: (i) to characterize the state of tree regeneration after mortality, (ii) to describe patterns of early post-disturbance reorganization relative to undisturbed reference conditions, and (iii) to assess what drives differences in forest reorganization patterns after disturbance. For the latter objective, we focused on the effect of active management (salvage logging, planting) versus no treatment (uncleared, unplanted), as well as on the effect of patch size, given that patch sizes are currently small in Central Europe but could increase under climate change. Based on a generally high recovery capacity of forests in Central Europe [23], we hypothesized that forests will regenerate well after mortality (as indicated by stem densities equal to or higher than those typically considered fully stocked in forest management), i.e. we did not expect to find indication of regime shifts away from forest ecosystems. For research questions 2 and 3, we quantified patterns of post-disturbance tree vegetation along the dimensions forest structure and composition, contrasting early post-disturbance stands to undisturbed reference conditions. We expected many disturbed forests to show early signs of change in tree species composition [26], here referred to as reassembly. Specifically, we expected the reassembly signal to be stronger for drought-prone Norway spruce forest types compared with other forest types, because of a declining competitiveness of the species in a warmer and drier world [27]. Furthermore, we expected a stronger reassembly signal on managed patches compared to unmanaged ones, as foresters actively adapt to a changing climate by planting new tree species. Management operations like salvage logging could also modify forest structure (here referred to as restructuring), by altering important niches for tree establishment via removing deadwood [28], or by damaging advance regeneration, which could impede tree recovery [29]. However, we generally expected a weaker signal of restructuring in post-disturbance forests compared with reassembly. We further hypothesized that disturbance patch size influences post-disturbance reorganization patterns [30]. The alternative hypothesis is that even large disturbance patches in Central Europe (i.e. several hectares in size) are still small enough to not limit seed dispersal, and that a high prevalence of advance regeneration will render the effect of patch size on post-disturbance forest recovery insignificant.

## 2. Method and materials

### (a) Study design

**Figure 1.**
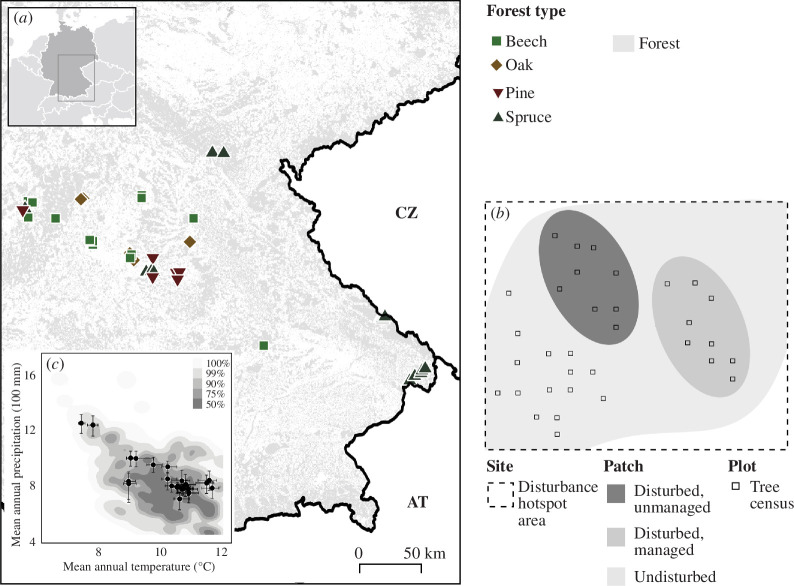
(*a*) Location of study sites in Central Europe and their pre-disturbance forest types. Sites were situated in disturbance hotspots in 2018–2020, as identified by remote sensing. (*b*) Each site consists of patches in three different treatments (disturbed unmanaged, disturbed managed and undisturbed reference) located in close proximity to each other (similar site and climate conditions), with tree censuses performed on sample plots within these patches at plot densities proportional to patch size. (*c*) The climatic conditions of the selected study sites (dots) represent the climatic conditions of all disturbance hotspots in Central Europe 2018–2020 (shaded, representing a probability density surface of the climate conditions across all disturbance hotspots) well. Whiskers represent the min and max value per site.

We surveyed sites in 2022 that experienced moderate- to high-severity tree mortality from drought and bark beetles between 2018 and 2020. We thus quantified early post-disturbance conditions, focusing on the tree regeneration already established a few years after disturbance. In a first step, areas of tree mortality were identified from remote sensing via the European forest disturbance map [31,32], selecting hotspot areas where annual excess mortality in 2018–2020 was >300% relative to the mean rate of canopy opening between 1986 and 2015. In a second step, we contacted forest owners and local forest authorities to locate sites that included in the immediate vicinity: (i) a disturbed patch that was unmanaged (standing and downed deadwood remaining on site), (ii) a disturbed patch that was managed (salvage-logged, often planted), and (iii) an undisturbed reference patch, representing the composition and structure prior to the onset of mortality in 2018 (figure 1). All selected sites were located in production forests that were regularly managed before 2018, and were in the optimal stage of stand development prior to disturbance. A detailed management history of the selected sites was not available, yet the three patches within each site were selected to not exhibit major differences in past treatment. The minimum patch size requirement for selection was 625 m², with the smallest side of the patch exceeding 25 m. We selected a total of 40 sites distributed throughout northern Bavaria, Germany, consisting of 120 patches in three treatments (disturbed and unmanaged, disturbed and managed, undisturbed reference). The elevation range of the sites was from 128 to 971 m.a.s.l. Mean annual temperature ranged between 7.5 and 11.5°C, and mean annual precipitation sum was between 750 and 1200 mm. The selected sites represent the climatic conditions of hotspots of recent mortality in Central Europe well (figure 1*c*). Average patch size was 6558 m², and thus close to the long-term average disturbance patch size in Germany of 7300 m² [31]. The largest patch extended over 22 500 m². Our sites included four major forest types: Norway spruce forests (*n* = 17 sites | 51 patches), Scots pine (*Pinus sylvestris* L.) forests (*n* = 5 | 15), European beech (*Fagus sylvatica* L.) forests (*n* = 13 | 39) and forests dominated by oak species (*Quercus robur* L. and *Quercus petraea* (Matt.) Liebl., *n* = 5 | 15). These four forest types jointly account for 73% of the forest area in Germany [33] and represent a wide range of disturbance response traits. Spruce has moderate to high shade tolerance and high susceptibility to drought and bark beetles. Spruce was widely cultivated across Central Europe for timber production, also in low-elevation areas outside of its native range, such as in the large majority of spruce forests under study here. Pine is a light-demanding species that has generally high drought tolerance; it often occurs on soils with low nutrient availability and water holding capacity in Central Europe. Beech is highly shade tolerant and moderately tolerant to drought; it would naturally dominate the low- to mid-elevation forests of Central Europe. Sessile and pedunculate oak are moderately light demanding species of high drought resistance; they naturally dominate in Central Europe where it is too dry or too wet for beech.

### (b) Data

For each of the 120 patches, sample plots were selected by randomly drawing locations from a regular 10 × 10 m grid overlaid over the patch area (electronic supplementary material, figure S1). For each plot, we collected data via two complementary sampling approaches, an area-based sampling and a distance-based sampling. In the area-based sampling, we recorded trees on 2 × 2 m plots. We deliberately chose a small plot size and corresponding higher number of replicates per patch because this allowed us to capture the spatial variation within patches, which is an important element in our analysis (see §2*c* below). We considered trees in three size cohorts: saplings (0.2–2.0 m in height), juveniles (from 2.0 m in height up to 10 cm in diameter at breast height [dbh]) and mature trees (>10 cm in dbh). Saplings and juveniles are henceforth jointly referred to as tree regeneration. Saplings were recorded in six height classes (0.2–0.4 m, 0.4–0.6 m, 0.6–0.8 m, 0.8–1.0 m, 1.0–1.3 m and 1.3–2.0 m). For juveniles and mature trees, dbh and height were recorded. In the distance-based sampling, we additionally measured the distance from the plot centre to the nearest juvenile and mature tree using a laser-based range finder (Nikon Forestry Pro II, Nikon Corp.). Maximum distance for distance-based sampling was 15 m from the plot centre. To robustly estimate stem densities for juveniles and mature trees (i.e. classes occurring with lower frequency than saplings), we calculated a second estimate of stem density for these classes from distance-based sampling, and averaged area- and distance-based estimates of stem density. Stem density for saplings was determined solely via area-based counts. Five to fifteen plots were sampled in each patch, with the number of plots increasing with patch size. At these sample sizes, the error rates of stem density estimates stabilized in simulations across a wide range of densities (see electronic supplementary material for details).

### (c) Analyses

In total, we analysed 1244 plots nested in 120 patches. We first investigated the state of the forest prior to disturbance (based on the 40 reference patches, holding 493 plots), and subsequently analysed post-disturbance recovery by forest type (80 disturbed patches with 751 plots). The central part of our analysis quantified patterns of post-disturbance forest reorganization, operationalizing the conceptual approach presented by Seidl & Turner [9] (see details below). Specifically, we conducted a site-level comparison of post-disturbance forest structure and composition to reference conditions. Disturbed patches were located in the immediate vicinity of reference patches (mean distance between patches within a site: 372 m) and were selected to have similar climate and soil conditions as well as forest management history; the signal derived from this site-level comparison thus controls for the influence of site conditions.

Forests are dynamic systems, and disturbances are the motor of forest development. Stem density, for instance, often decreases in a disturbance, but rises sharply a few years later (when the next generation of trees has responded successfully to the increased resource availability after disturbance), only to decrease gradually over the coming decades due to competition-induced mortality (‘self-thinning’). Post-disturbance reorganization, however, can also respond to drivers of global change, setting forests on a different development trajectory (e.g. reducing the rate and success of tree establishment, and hence leading to reduced competition between trees and more open forest structures). Here, we were interested in isolating the latter (i.e. an early post-disturbance signal of change in forest dynamics; henceforth referred to as forest change), from the historical pattern of post-disturbance forest development that leads back to the conditions observed in undisturbed reference patches (table 1). The historical pattern of post-disturbance stand development was informed by observations on past post-disturbance recovery in combination with observed conditions in our undisturbed reference patches, and represents post-disturbance forest resilience *sensu stricto*. It assumes that disturbed sites have higher stem density in the regeneration a few years after disturbance but lower structural diversity in both vertical and horizontal structure (due to mortality removing the overstory and tree regenerating filling in gaps either naturally or by planting). Furthermore, it assumes the dominant species pre-disturbance to self-replace under historical post-disturbance stand development (either by planting or natural regeneration), with higher tree species richness and lower community-weighted shade tolerance post-disturbance because of the ephemeral presence of early-seral species (table 1).

**Table 1.** Assessing post-disturbance reorganization. The six indicators assessed, their measurement and the discrimination between stand development consistent with observed reference conditions (i.e. resilience *sensu stricto*) and patterns that indicate post-disturbance forest change.

dimension	indicator	stand development towards reference conditions (i.e. resilience *sensu stricto*)	indication for post-disturbance forest change	measurement
structure	stem density (S1)	post-disturbance stem densities equal to or exceeding undisturbed reference values, eventually undergoing stem exclusion and self-thinning; reference stem densities can be reached based on the post-disturbance stem density	post-disturbance stem densities are lower than undisturbed reference stem densities	stem density (N ha^−1^)
	horizontal structure (S2)	the post-disturbance forest has fewer and/ or smaller gaps than undisturbed reference conditions, with stands regenerating homogeneously after disturbance and gaps being created only in the later stages of stand development	the post-disturbance forest has more and/ or larger gaps than under undisturbed reference conditions	average distance (m) from plot centre to the nearest juvenile or adult tree
	vertical structure (S3)	the vertical complexity of forests post-disturbance is lower than under undisturbed reference conditions; vertical complexity increases with stand development	the vertical complexity of forests post-disturbance is as high or higher than under undisturbed reference conditions	number of vertical layers present (saplings, juveniles, mature trees)
composition	dominant species (C1)	the species dominating under undisturbed reference conditions also dominates post-disturbance; the dominant species is self-replacing.	the dominant species under undisturbed reference conditions has lower importance post-disturbance	relative importance value^a^ of the species that dominated under reference conditions
	tree species richness (C2)	tree species richness is higher post-disturbance compared to undisturbed reference conditions, making it likely that reference richness will re-emerge, given competitive exclusion during stand development.	tree species richness is lower post-disturbance compared to undisturbed reference conditions, making it unlikely that the reference tree species richness is reached again	tree species richness
	competition (C3)	resource competition post-disturbance is lower than under undisturbed reference conditions	resource competition post-disturbance is higher than under reference conditions	community-weighted mean shade tolerance^b^

^a^ Relative importance value of the most dominant species was calculated based on basal area (juveniles and adult trees) and stem density (all three cohorts), and scaled between 0 and 100. Higher values indicate stronger dominance.

^b^ Shade-tolerance rating based on Niinemets & Valladares [34], with one being light demanding and five highly shade tolerant.

We quantified forest reorganization patterns along the two dimensions forest structure (i.e. the number, size and spatial arrangement of trees) and forest composition (i.e. the identity and diversity of tree species) [9]. We investigated three indicators of forest composition and three indicators of forest structure (see table 1 for details). To determine whether there is a deviation from a trajectory towards reference conditions in a given indicator, we related differences in the patch-level mean to the variation inherent in the system. Specifically, we standardized all indicators by dividing the observed differences (post-disturbance mean versus mean under reference conditions) by the standard deviation of the indicator under reference conditions, derived from all plots on a given patch. The three indicators for each dimension were aggregated via a maximum operator, emphasizing the indicator that gives the strongest signal of change per dimension. We selected the maximum over the mean for indicator aggregation as averaging can result in compensation between indicators (i.e. a low indicator value offsetting a high indicator value), potentially masking the signal of change we were interested in. We plotted the aggregated indicators in an orthogonal state space, where axes represent change in composition versus change in structure. The signal strength of reorganization (RI) at site level was calculated as the Euclidian distance to zero in this orthogonal space, with higher values indicating a stronger trajectory away from resilience.

We considered the system to be resilient *sensu stricto* (i.e. no deviation from a trajectory towards reference conditions) when RI was equal to or lower than one standard deviation. For values of RI > 1, we also assessed the pattern of reorganization (i.e. reassembly, restructuring and replacement) by determining the directionality of change from its two components structural and compositional change. If deviations in structure dominate (i.e. structural change ≥ tan(60°) times compositional change, i.e. all points situated above a 60° incline in orthogonal space of structural change~compositional change), we refer to the pattern as restructuring, while the dominance of compositional changes (with structural change < tan(30°) times compositional change, i.e. all points below a 30° incline) indicates reassembly. A simultaneous deviation of forest structure and composition from reference conditions (i.e. points located between 30° and 60° in an orthogonal space of structural change~compositional change) indicates a trajectory towards replacement of the previous system with a structurally and compositionally different post-disturbance system. For all three categories, we assumed 1 < RI ≤ 2 to indicate moderate reorganization away from resilience, while RI > 2 suggest strong reorganization. See electronic supplementary material, tables S1–S4 for examples illustrating the detailed calculation of indicator values, RI and reorganization patterns. To test whether the strength of the reorganization signal differs between managed and unmanaged patches, we used Wilcoxon’s signed rank sum test. Linear regression was used to investigate the relationship between patch size and RI.

## 3. Results

### (a) Post-disturbance structure and composition

Undisturbed reference stands had a median stem density of 1002 mature trees (>10 cm dbh) per hectare. Beech forests had the lowest (731 stems ha^−1^) and pine forests the highest stem densities (1163 stems ha^−1^). Stands were characterized by ample advance regeneration prior to disturbance, with 11 954 and 1040 stems ha^−1^ in the sapling and juvenile cohorts, respectively. Spruce forests had the lowest densities of advance regeneration (5141 and 472 stems ha^−1^), while beech forests had the highest (17 640 and 2873 stems ha^−1^). Stands had a complex vertical structure (median number of layers of 2.7) but a simple horizontal structure, being closed with little gaps (median distance to nearest juvenile or adult tree 3.5 m). Stands were generally strongly dominated by a single species (median relative IV value of the single most dominant species 65.9%), and patch-level species richness was moderate, with 5.0 tree species per patch and 2.0 tree species per plot (median values).

The median disturbance severity expressed in percent of basal area of mature trees killed was 66.8%. Severity was highest in spruce forests (81.0%) and lowest in oak forests (36.8%). Stem density in the regeneration layer post-disturbance (median of 11 897 stems ha^−1^) was comparable with undisturbed reference values (table 2). No patch was completely unstocked after disturbance, and stem densities were considerable even for patches with the lowest stocking levels (minimum stem density in the regeneration: 15 stems ha^−1^, fifth percentile: 1172 stems ha^−1^). Regeneration density was generally high across all forest types, ranging from 9927 stems ha^−1^ in oak forest types to 14 978 stems ha^−1^ in beech forest types. Regeneration post-disturbance was largely horizontally homogeneous (median distance to the next juvenile or mature trees of 2.1 m). The moderate disturbance severity and high prevalence of advance regeneration also resulted in high vertical complexity post-disturbance (median number of layers 2.6). Dominance values for the most prominent species were lower than under undisturbed reference conditions (median: 50.8%), but tree species richness was similar (median of 2.1 tree species per plot and 5.0 tree species per patch). Consistent with the high prevalence of advance regeneration, the community-weighted shade tolerance of regeneration post-disturbance was high (median of 4.0 on a relative scale where 1 is light demanding and 5 is highly shade tolerant).

**Table 2.** Structure and composition of forested areas in Central Europe disturbed in 2018–2020. Values are medians across all disturbance patches, with the 5th–95th percentile range of patch-level values indicated in parenthesis. *n* = number of disturbance patches | number of sample plots analysed.

		forest type				all (*n* = 80 | 751)
		Norway spruce (*n* = 34 | 351)	Scots pine (*n* = 10 | 67)	European beech (*n* = 26 | 261)	Oak ssp. (*n* = 10 | 72)	
structure	S1: stem density (stems ha^−1^)	10 493 (1826–33 624)	13 212 (2132 – 46 146)	14 978 (1715–31 111)	9927 (1297–67 833)	11,897 (1172–40 092)
	S2: horizontal structure (m)	3.24 (1.31–5.82)	1.71 (1.08–3.34)	1.30 (0.70–4.80)	1.83 (1.13–2.53)	2.12 (0.83– 5.32)
	S3: vertical structure (no. of layers)	2.40 (1.59–2.91)	2.72 (1.18– 3.00)	2.60 (1.90–3.00)	2.80 (2.09–3.00)	2.55 (1.60– 3.00)
composition	C1: dominant species (rel. IV)	37.8 (0.00–77.8)	45.1 (6.9– 93.7)	80.4 (16.5–99.4)	47.1 (4.2– 92.6)	50.8 (0.2– 97.9)
	C2: species richness (sp. per 4 m²)	2.2 (1.5–3.3)	2.3 (1.0–3.2)	1.5 (1.0–3.6)	2.3 (1.3–3.3)	2.1 (1.1–3.6)
	C3: competition (dim.)	4.1 (2.7–4.5)	2.7 (2.0–4.2)	4.3 (3.5–4.6)	2.8 (2.6–3.5)	4.0 (2.6–4.6)

### (b) Post-disturbance forest reorganization

**Figure 2.**
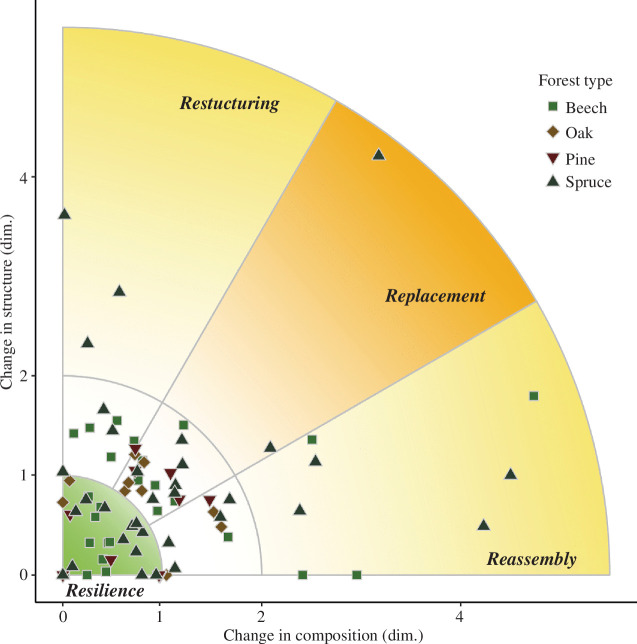
Pattern and strength of the reorganization signal in post-disturbance forests of Central Europe. dim. = dimensionless.

**Figure 3.**
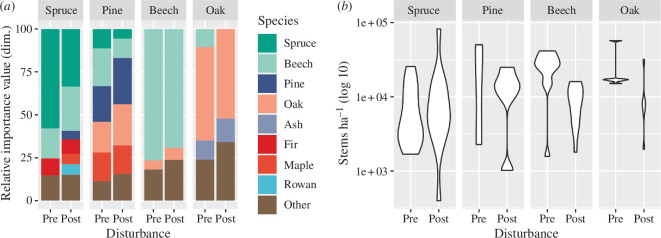
Disturbance-mediated change in (*a*) species composition and (*b*) stem density in the four main forest types analysed. Values are shown for plots that (*a*) undergo reassembly or replacement, and (*b*) experience restructuring or replacement. Pre = undisturbed reference conditions, post = post-disturbance conditions.

Overall, 36.3% of patches were resilient (i.e. on a development trajectory to reference conditions), while 63.7% showed indication of forest change post-disturbance (RI > 1), with a strong reorganization signal (RI > 2) on 16.3% of patches (figure 2). Replacement was the most common pattern of reorganization (43.1% of all patches showed signs of change in both structure and composition), followed by reassembly (33.4%) and restructuring (23.5%). However, among the patches showing a strong reorganization signal, reassembly was the dominant pattern (61.5% of patches with RI > 2). Generally, the signal strength of reorganization was higher for forests undergoing reassembly (RI = 2.41) compared with those experiencing restructuring (RI = 1.83) and replacement (RI = 1.48). Only spruce and beech forest types showed a strong signal of reorganization away from resilience, with spruce forests accounting for 69.2% of all patches with RI > 2. Of all spruce patches investigated, only 35.3% were assessed to be resilient (i.e. with no signs of change). Also, the average strength of the reorganization signal was highest in spruce forest types (RI = 1.70), followed by beech (RI = 1.37), oak (RI = 1.22) and pine (RI = 1.03) forest types. Nonetheless, due to the strong influence of advance regeneration, 82.4% of all spruce forest types still had spruce as the dominant species in the regeneration layer post-disturbance. The indicator contributing most to a signal of compositional change was a reduction in the importance value of the tree species dominating under reference conditions. The indicator having the strongest influence on changes in forest structure was a reduction in stem density (see figure 3).

### (c) Effects of management and patch size

**Figure 4.**
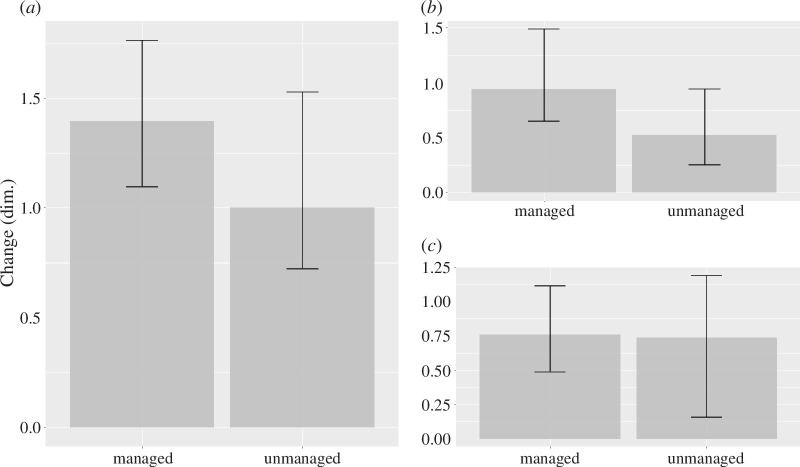
(*a*) The effect of forest management on the signal strength of post-disturbance reorganization, and the two components of reorganization, (*b*) change in composition and (*c*) change in structure. Shown are median values (bars) and interquartile ranges (whiskers) across all forest types. dim. = dimensionless.

The share of resilient patches was considerably lower in managed forests (22.5%) compared with forests without post-disturbance management (50.0%); in other words: management facilitates change. Reassembly and replacement more than doubled (from 12.5% to 30.0%, and from 17.5% to 37.5%) in managed stands compared with unmanaged stands, while patches undergoing restructuring decreased (from 20% under unmanaged conditions to 10% under management). Management also increased the strength of the reorganization signal by +0.59 (*p* = 0.0051, Wilcoxon rank sum test). It mainly facilitated changes in composition (+0.60, *p* = 0.0005) rather than structure (+0.16, *p* = 0.553; figure 4). The effect of management was strongest in spruce forests (+0.82, *p* = 0.080), where it was driven by distinct changes in forest composition (+0.87, *p* = 0.0093). Patches that were resilient (i.e. no change post-disturbance) were smaller (median patch size 3500 m²) than those undergoing reassembly (6000 m²) or replacement (5300 m²). Furthermore, reorganization signal strength increased significantly with size of the disturbance patch (*p* = 0.027, linear regression model), with RI increasing by 0.48 for each additional hectare in patch size.

## 4. Discussion and conclusions

### (a) Hypotheses revisited

Here, we presented an approach to quantify patterns of forest reorganization across multiple indicators of forest structure and composition, following a widespread pulse of tree mortality in Central Europe. Our analyses revealed evidence for changes in forest composition, while post-disturbance stem densities were generally high already a few years after disturbance. Notably, we found no indication of ongoing forest loss across the four dominant forest types analysed. This is consistent with other studies investigating post-disturbance forest recovery both empirically [18,35–37] and by means of remote sensing [10,23,38]. A widespread regime shift away from forest ecosystems is thus unlikely under the conditions currently prevailing in Central Europe. We also found that advance regeneration plays a prominent role in post-disturbance reorganization in our study area. Previous studies have highlighted the role of advance regeneration in Central Europe, indicating that the cohort of trees dominating post-disturbance stand development established prior to disturbance [19–21]. Contrastingly, other studies from the region reported that the majority of trees regenerate only after disturbance, with little or no advance regeneration [35,36]. In the context of drought, a particular benefit of advance regeneration might be that subcanopy trees are buffered from climatic extremes when they establish; they experience a more moderate microclimate [39] compared with canopy trees or trees regenerating on areas where the canopy has been removed. We note, however, that the importance of advance regeneration for post-disturbance forest development is strongly contingent on the disturbance agent at play (here: drought and bark beetle disturbances, which do not directly impact advance regeneration). Disturbances such as windthrow (disturbing the soil via uprooting) and wildfire (disturbing the forest floor via heat and combustion) leave distinctly different disturbance legacies, and could result in alternative trajectories of post-disturbance forest development [37,40].

Despite the prominent role of disturbance legacies in the form of advance regeneration, we found a robust signal of post-disturbance forest change. Only approximately one-third of the analysed patches were resilient *sensu stricto* (i.e. with forest structure and composition on a trajectory back to reference conditions; cf. table 1). Given the high prevalence of advance regeneration, this finding suggests that the observed changes predate the disturbance process, reflecting dynamics underway already for many years (e.g. where beech is invading closed-canopy spruce forests, or oak is regenerating under the canopy of pre-disturbance pine forests). In line with our hypothesis, we found particularly strong indication for post-disturbance change in spruce forest types. This underlines that—in addition to being the forest type most severely disturbed—both ongoing climate change and foresighted silviculture are reducing the prevalence of spruce in Central Europe [17]. A more surprising finding was that also a considerable share of beech forests are undergoing change. The vulnerability of beech regeneration to drought was recently highlighted by other research [41]. As a consequence, the dominance of beech—which was historically the most competitive species in Central Europe—might decrease under climate change. We also found support for a forest reorganization response to increasing size of disturbance patches. Given that patch sizes are generally small in Central Europe compared with the dispersal abilities of tree species, and given the prominent role of advance regeneration in post-disturbance development, this signal is most likely related to the climatically more extreme conditions found on larger patches [39], rather than being an effect of dispersal limitation [42].

### (b) Methodological considerations

Studying the short time window after disturbance during which forests reorganize has considerable potential to better understand the processes that foster or impede forest recovery. We here present the first application of a recently developed concept to assess and quantify patterns of forest reorganization with the aim to better understand disturbance-mediated forest change [9]. Specifically, our approach is characterized by: (i) defining reference conditions against which to assess change, (ii) quantifying change based on a set of indicators describing forest structure and composition, and (iii) evaluating change against patterns of post-disturbance forest development leading back to reference conditions (cf. table 1). We here used undisturbed mature forests as reference condition; other reference values, such as desired system states and fluxes [43] or the potential natural vegetation [44], would result in different outcomes. Furthermore, while we selected undisturbed reference patches with utmost care, uncertainties remain regarding their representativeness in terms of stand conditions (are reference conditions a true equivalent to disturbed patches prior to disturbance?) and site conditions (do reference patches and disturbed patches experience similar climate and soil conditions?). We aimed to minimize these uncertainties by selecting reference sites in very close proximity to disturbed sites (mean distance between patches: 372 m), and by consulting with local forestry personnel on the stand conditions of disturbed patches prior to disturbance. We furthermore considered a disturbance-induced change in an indicator relative to its inherent spatial variation (relating the mean change to the standard deviation of the indicator), acknowledging that ecosystems are inherently variable and that only a deviation that exceeds this inherent variation of the system will constitute true change. We note that we opted for relatively small (but many) study plots, resulting in a high level of spatial variation in our data. Consequently, a reorganization signal of two standard deviations is likely to indicate substantial ecological change.

An important hallmark of our analysis is that we evaluated change against patterns of post-disturbance development leading back to reference conditions. This acknowledges that forests change continuously throughout forest development, and reflects our focus on signs of deviation from these trajectories of resilience. We note that assessing forests 2–4 years after disturbance can only provide an early indication of post-disturbance development, given that recovery intervals may extend over decades [23]. Currently existing gaps in tree regeneration can still fill in at a later point, and new species might still colonize disturbed patches [45]. Any such protracted changes will also leave an imprint on forest structure and composition (e.g. increased structural heterogeneity through gradual infilling of gaps [38]). We note that our assessment of forest change is thus conservative (i.e. even if we do not find indication of change currently, the system might still change later). Conversely, where we already see considerable signs of change, it is very unlikely that the system will return to a trajectory towards the reference state. Also, given the high horizontal homogeneity of the regeneration and the high community-weighted shade tolerance of the already established trees in our system, the current cohort of trees will likely dominate forest development for at least several decades (i.e. time frames of relevance for forest policy and management). This notion is supported by recent analyses based on remote sensing data, highlighting that the long-term recovery trajectory of forests can be successfully predicted from the state of the system 3 years after disturbance [10]. Nonetheless, the potential of early post-disturbance analyses—such as the one conducted here—to serve as early warning indicators of forest change should be further investigated in future research.

### (c) Implications and conclusions

By operationalizing a recently developed concept of forest reorganization, we provide a quantitative and nuanced perspective on the strength and direction of early post-disturbance forest change. Disturbances are strong agents of forest change, and the increasing frequency of disturbances under climate change in Central Europe [12,31], thus creates opportunities to adapt to emerging novel climatic conditions. Our findings of compositional changes especially in spruce forest types are consistent with reports of a thermophilization of vegetation in many parts of the globe [46,47]. Conversely, a shift towards more open forests—also expected to be a widespread response of forests to warmer and drier conditions [4]—is not evident in our data. Rather, our analyses suggest that stem density and horizontal structure of the next cohort of trees will be largely similar to the previous cohort. These findings imply that disturbances act as catalysts of forest change in Central Europe, but that these changes are only partly consistent with globally expected patterns.

The finding that the signal of reassembly is stronger in managed versus unmanaged forests clearly demonstrates that management in Central Europe is actively altering tree species composition, e.g. in order to adapt forests to climate change [48]. In line with current scientific understanding and forest policy, the strongest effect of management was detected for spruce forests, which—after being promoted heavily for 150 years—are now the main focus of conversion activities in Central Europe [49,50]. Conversely, we found that management did not substantially change forest structure. Only 2–4 years after disturbance, stem densities were already high, and only 12.8% of all unmanaged patches had stem densities below 2500 stems ha^−1^ (i.e. common target densities for tree planting in Central Europe). Our data thus suggest that while post-disturbance tree planting can be an important tool to accelerate desired species change, it is currently not strictly needed to sustain forest cover in Central Europe. We note that resilience *sensu stricto*—here used to describe a trajectory returning to reference conditions—is not *per se* good or bad. In the context of the spruce forests of lowland Central Europe, for instance, a reorganization towards more climate-adapted species assemblages is desirable in order to maintain many forest functions and services. The fact that these forests were not found to be resilient is thus a positive result from the point of view of forest management.

Our finding of a prominent role of advance regeneration in forests affected by drought and bark beetles has important implications for forest management. Specifically, it highlights that the conditions that determine post-disturbance forest reorganization were already set well in advance of the disturbance event. Opening the canopy early in stand development to facilitate structural complexity via the development of advance regeneration can be beneficial for post-disturbance recovery [51]. Advance regeneration developing underneath existing canopies largely consists of shade-tolerant species, however, while many light-demanding early-seral species have broader fundamental niches and might be better able to cope with increasing climatic extremes [34,48]. Furthermore, it remains unclear how advance regeneration—which has been sheltered from climatic extremes in the subcanopy—will fare under future extremes, now that the disturbance of 2018–2020 has removed their canopy shelter. We conclude that the recent wave of disturbances is changing the forests of Central Europe, and that studying post-disturbance forests can give insights into the patterns and processes of this ongoing reorganization process.

## Data Availability

All underlying data and derived data pertinent to the results presented here as well as the code used for analyses are available on Figshare at [52]. All software used for this work is properly cited and publicly available. Supplementary material is available online [53].
